# Fluorescence-Guided Surgery of Retroperitoneal-Implanted Human Fibrosarcoma in Nude Mice Delays or Eliminates Tumor Recurrence and Increases Survival Compared to Bright-Light Surgery

**DOI:** 10.1371/journal.pone.0116865

**Published:** 2015-02-24

**Authors:** Fuminari Uehara, Yukihiko Hiroshima, Shinji Miwa, Yasunori Tome, Shuya Yano, Mako Yamamoto, Yasunori Matsumoto, Hiroki Maehara, Kazuhiro Tanaka, Michael Bouvet, Fuminori Kanaya, Robert M. Hoffman

**Affiliations:** 1 AntiCancer, Inc., 7917 Ostrow Street, San Diego, California 92111, United States of America; 2 Department of Surgery, University of California San Diego, 200 West Arbor Drive, San Diego, California 92103, United States of America; 3 Department of Orthopedic Surgery, Graduate School of Medicine, University of the Ryukyus, 207 Uehara, Nishihara, Okinawa 903-0125, Japan; National Cancer Institute, UNITED STATES

## Abstract

The aim of this study is to determine if fluorescence-guided surgery (FGS) can eradicate human fibrosarcoma growing in the retroperitoneum of nude mice. One week after retroperitoneal implantation of human HT1080 fibrosarcoma cells, expressing green fluorescent protein (GFP) (HT-1080-GFP), in nude mice, bright-light surgery (BLS) was performed on all tumor-bearing mice (n = 22). After BLS, mice were randomized into 2 treatment groups; BLS-only (n = 11) or the combination of BLS + FGS (n = 11). The residual tumors remaining after BLS were resected with FGS using a hand-held portable imaging system under fluorescence navigation. The average residual tumor area after BLS + FGS was significantly smaller than after BLS-only (0.4 ± 0.4 mm^2^ and 10.5 ± 2.4 mm^2^, respectively; p = 0.006). Five weeks after surgery, the fluorescent-tumor areas of BLS- and BLS + FGS-treated mice were 379 ± 147 mm^2^ and 11.7 ± 6.9 mm^2^, respectively, indicating that FGS greatly inhibited tumor recurrence compared to BLS. The combination of BLS + FGS significantly decreased fibrosarcoma recurrence compared to BLS-only treated mice (p < 0.001). Mice treated with BLS+FGS had a significantly higher disease-free survival rate than mice treated with BLS-only at five weeks after surgery. These results suggest that combination of BLS + FGS significantly reduced the residual fibrosarcoma volume after BLS and improved disease-free survival.

## Introduction

Most tumors in the retroperitoneum are malignant, and about one third of these are soft tissue sarcomas [1, 2]. Retroperitoneal tumors present several therapeutic challenges because of their relative late presentation and anatomical location [3]. Complete tumor resection can potentially be a curative treatment modality for retroperitoneal soft tissue sarcoma patients [4], but local recurrence occurs in a large proportion of patients and is responsible for as many as 75% of sarcoma-related deaths [5].

Local recurrence often occurs following attempted curative resection of the primary tumor, because all cancer cells are not removed by the surgeon due to the inability to see them. Making tumors fluorescence offers great advantages for fluorescence-guided surgery (FGS) to achieve complete resection [6].

Our laboratory has developed FGS of cancer using both fluorescent-protein labeling of the tumor [7–16] as well as fluorescent-antibody labeling of the tumor [17–29], in orthotopic nude mouse models of human tumors, including patient-derived orthotopic (PDOX) models [24, 25, 30].

In the present study, we report the effectiveness of using FGS to improve outcomes in a retroperitoneal-implanted nude-mouse model of human fibrosarcoma, including reducing residual tumor tissue, thereby decreasing tumor recurrence and increasing disease-free survival.

## Materials and Methods

### Ethics Statement

All animal studies were conducted with an AntiCancer Institutional Animal Care and Use Committee (IACUC)-protocol specifically approved for this study and in accordance with the principals and procedures outlined in the National Institute of Health Guide for the Care and Use of Animals under Assurance Number A3873-1. In order to minimize any suffering of the animals the use of anesthesia and analgesics were used for all surgical experiments. Animals were anesthetized with a 20 μL mixture of ketamine (20 mg/kg), acepromazine (0.48 mg/kg), and xylazine (15.2 mg/kg) by intramuscular injection 10 minutes before surgery. The response of animals during surgery was monitored to ensure adequate depth of anesthesia. Ibuprofen (7.5 mg/kg orally in drinking water every 24 hours for 7 days post-surgery) was used in order to provide analgesia post-operatively in the surgically-treated animals. The animals were observed on a daily basis and humanely sacrificed by CO_2_ inhalation when they met the following humane endpoint criteria: prostration, skin lesions, significant body weight loss, difficulty breathing, epistaxis, rotational motion and body temperature drop. The use of animals was necessary to understand the in vivo efficacy, in particular, anti-metastatic efficacy of the procedures tested. Animals were housed with no more than 5 per cage. Animals were housed in a barrier facility on a high efficiency particulate air (HEPA)-filtered rack under standard conditions of 12-hour light/dark cycles. The animals were fed an autoclaved laboratory rodent diet (S1 ARRIVE Checklist).

### Establishment of a green fluorescent protein labeled HT-1080 fibrosarcoma cell line

For green fluorescent protein (GFP) gene transduction of human HT-1080 fibrosarcoma cells [31, 32], 80% confluent cells were used. Briefly, cells were incubated with a 1:1 precipitated mixture of retroviral supernatants, of packaging PT67-GFP cells [33–35], which express the GFP gene linked to the G418 resistance gene (Clontech, Mountain View, CA) and RPMI 1640 medium (Cellgro, Herndon, VA, USA) containing 10% fetal bovine serum (FBS) (Omega Scientific, San Diego, CA, USA) for 72 h. Fresh medium was replenished at this time. Cells were harvested with trypsin/EDTA 72 h post-transduction and subcultured at a ratio of 1:15 into medium, which contained 200 μg/ml of the selective agent G418. The level of G418 was increased stepwise up to 800 μg/ml [33–35].

### Cell culture

HT1080-GFP cells were maintained in RPMI 1640 medium supplemented with 10% FBS and 1% penicillin/streptomycin. The cells were incubated at 37°C in a humidified atmosphere of 5% CO_2_ in air and harvested by trypcinization at 80% confluence.

### Mice

Athymic nu/nu nude mice (AntiCancer Inc., San Diego, CA) were used in this study. Mice were kept in a barrier facility under HEPA filtration. Mice were fed with an autoclaved laboratory rodent diet. All mouse surgical procedures and imaging were performed with the animals anesthetized by subcutaneous injection of the ketamine mixture described above (20 μl).

All animal studies were conducted with an AntiCancer Institutional Animal Care and Use Committee (IACUC)-protocol specifically approved for this study and in accordance with the principals and procedures outlined in the National Institute of Health Guide for the Care and Use of Animals under Assurance Number A3873-1.

### Retroperitoneal implantation of HT1080-GFP cells

Four-week-old female nude mice were anesthetized by the ketamine mixture via s.c. injection. The back was sterilized with alcohol. An approximately 1 cm skin incision was made just to the right side of spine in order to expose the retroperitoneum. HT1080-GFP cells (1×10^6^) in Matrigel (5 μl) (BD Bioscience, San Jose, CA) per mouse were injected into the retroperitoneum with a 0.5 ml 28 G latex-free insulin syringe (TYCO Health Group LP, Mansfield, MA). The skin was closed with a 6-0 suture.

### Fluorescence imaging

The Olympus OV100 Small Animal Imaging System (Olympus Corp., Tokyo, Japan), containing an MT-20 light source (Olympus Biosystems, Planegg, Germany) and DP70 CCD camera (Olympus Corp., Tokyo, Japan) [36]; the Dino-Lite imaging system (AM4113T-GFBW Dino-Lite Premier; AnMo Electronics Corporation, Taiwan) [30]; and the MVX10 long-working distance microscope (Olympus Corp.) [37] were used for imaging live mice. To analyze for recurrence and to follow tumor progression postoperatively, weekly noninvasive whole-body imaging of the mice was performed using the iBox Scientia Small Animal Imaging System (UVP LLC, Upland, CA, USA). GFP fluorescent-tumor areas were recorded every week [16, 38–40]. The working-distance setting for GFP imaging in the iBox was adjusted to image the maximum possible fluorescent area in each mouse both before surgery and after surgery. All images were analyzed with ImageJ v1.440 (National Institutes of Health).

### Tumor resection

**Figure 1:**
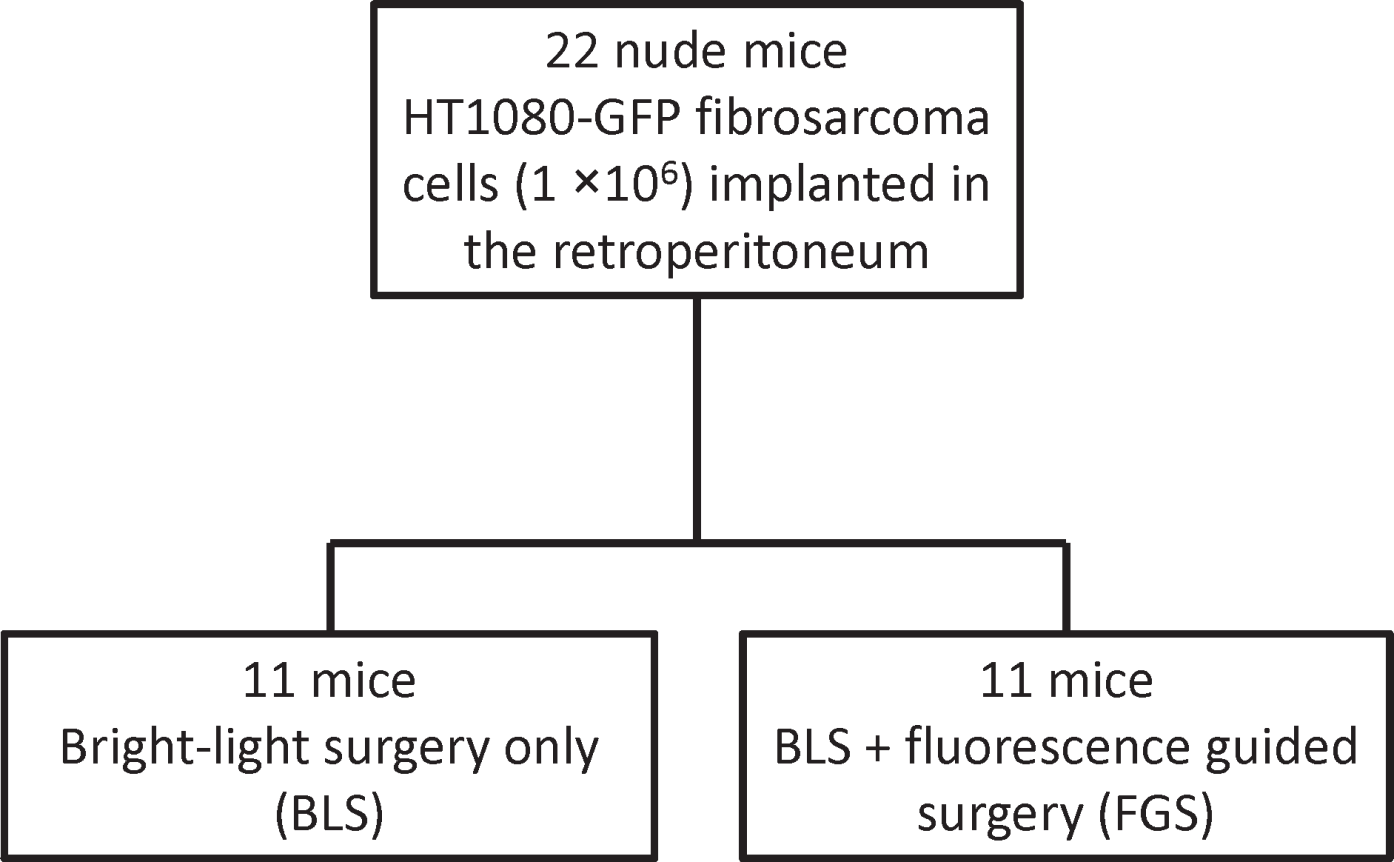
Schematic diagram of the experimental protocol. One week after retroperitoneal fibrosarcoma implantation in 22 mice, the tumor-bearing mice were randomly assigned to either the BLS-only or BLS + FGS groups. Resection of the tumor was performed using the Dino-Lite imaging system under fluorescence navigation (14). Postoperatively, the surgical resection bed was imaged with the OV100 at a magnification of 0.14× or 0.56× to detect residual fibrosarcoma cells.

One week after retroperitoneal implantation of HT1080-GFP cells, bright-light surgery (BLS) was performed to all tumor-bearing mice (n = 22). The exposed retroperitoneal tumor was imaged preoperatively with the OV100 at a magnification of 0.14×. Resection of the primary tumor was performed under standard bright-field using the MVX10 microscope [37]. For fibrosarcoma resection, intralesional and marginal tumor excision was performed in all the mice. Postoperatively, the surgical resection bed was imaged with the OV100 at a magnification of 0.14× or 0.56× to detect residual tumor. The mice which underwent BLS were randomized into 2 treatment groups: BLS only (n = 11) and BLS + FGS (n = 11) (Fig. 1). The residual tumors of the BLS + FGS group of mice were resected using the Dino-Lite imaging system under fluorescence navigation. After completion of FGS, the surgical resection bed was imaged with the OV100 at a magnification of 0.14× or 0.89× to detect microscopic minimal residual cancer (MRC) [41]. The incision was closed in one layer using 6-0 nylon surgical sutures after treatment.

### Statistical analysis

Statistical analyses were performed with EZR (Saitama Medical Center, Jichi Medical University). Residual tumor area is expressed as mean ± SD. The two-tailed Student’s t-test was used to compare continuous variables between 2 groups. Kaplan-Meier survival curves were used for demonstrating mouse survival. Survival outcomes were compared using log rank tests. A p value < 0.05 was considered statistically significant.

## Results and Discussion

### Efficacy of bright-light and bright-light plus fluorescence-guided surgery

**Figure 2:**
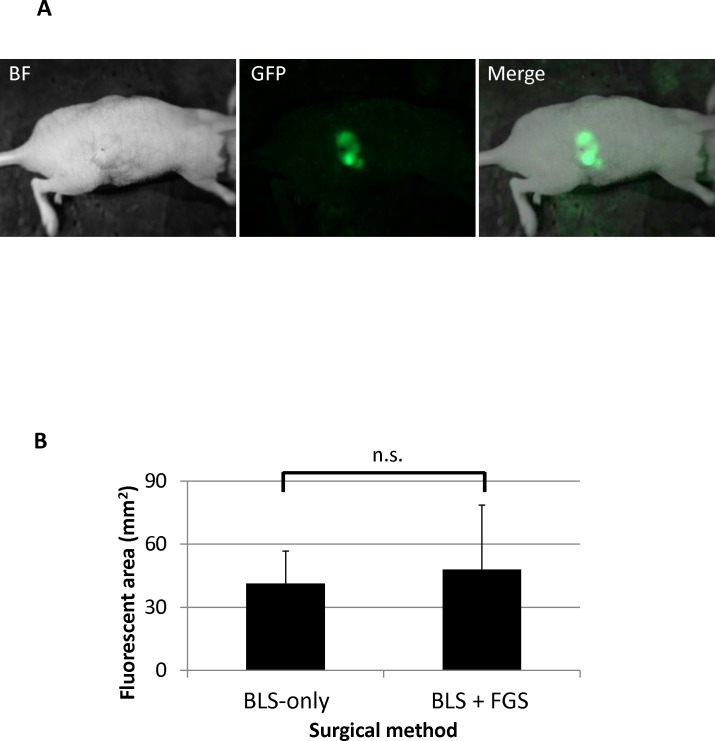
Pre-operative images of retroperitoneal fibrosarcoma mouse model. A. Retroperitoneal fibrosarcoma mouse model. B. Fluorescent-tumor areas before surgery. There was no significant difference in fluorescent-tumor areas between the BLS-only and BLS + FGS mouse groups. The fluorescent areas of tumors are expressed mean ± SD. All residual tumors expressing GFP were imaged with the Olympus OV100 Small Animal Imaging System (Olympus Corp.) [36] and analyzed with ImageJ v1.440 (National Institutes of Health). Statistical analysis was performed using the Student’s t-test.

**Figure 3:**
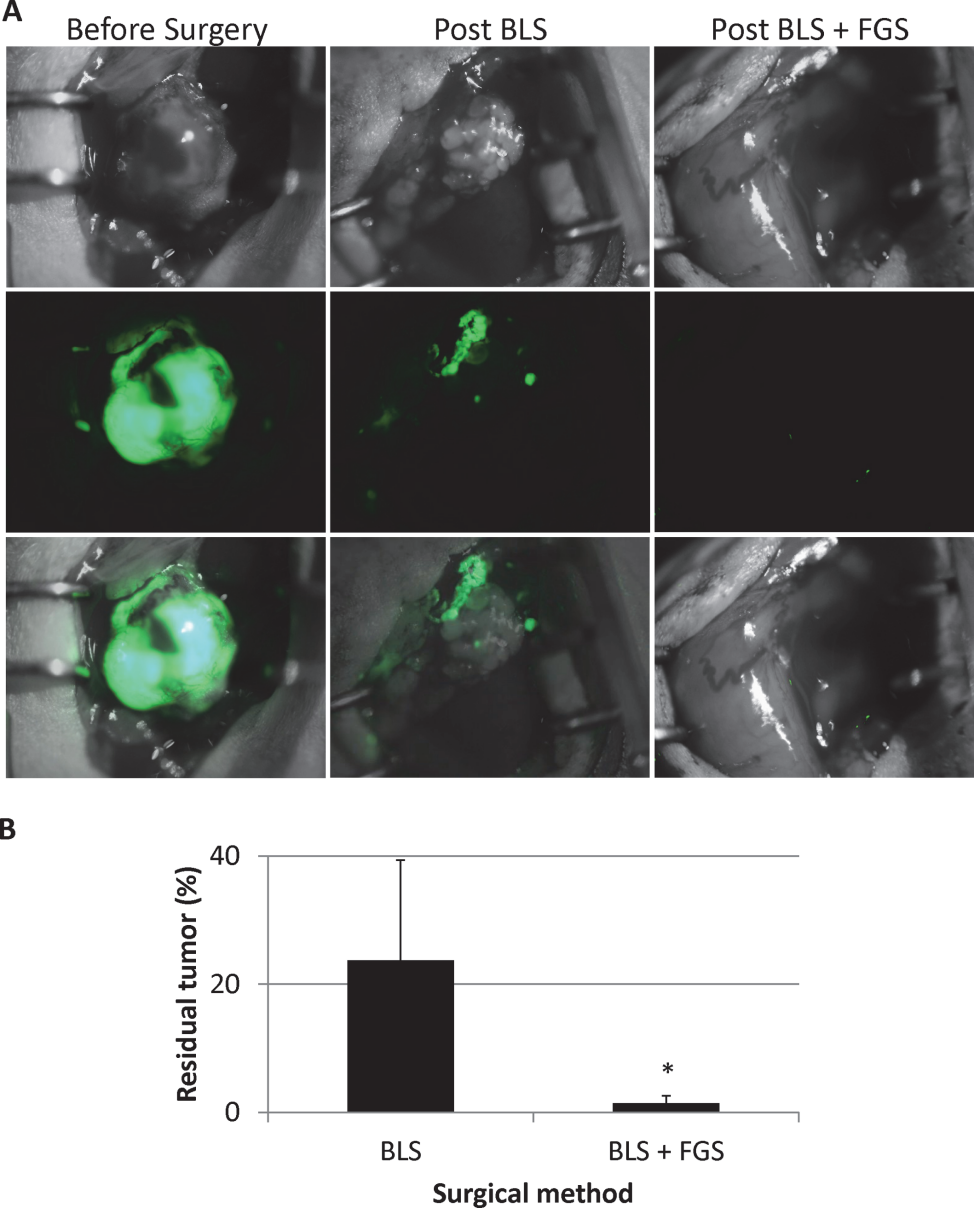
Pre-operative and post-operative images of retroperitoneal fibrosarcoma. A. Pre- and post-operative images of mice in the BLS-only and BLS + FGS groups. Upper panels are bright-field (BF), middle panels show GFP tumor fluorescence, and lower panels show merged images. The residual tumor after BLS-only was clearly detected with the OV100 at a magnification of 0.56×. The residual tumor after BLS + FGS was marginally detected with the OV100. Fluorescent area of residual tumor after BLS-only and BLS + FGS. Fluorescent areas of residual tumors in BLS-only and BLS + FGS group were 10.5 ± 2.4 mm^2^ and 0.4 ± 0.4 mm^2^, respectively. The residual tumor area after BLS + FGS was significantly smaller than after BLS-only. All images were measured for residual tumor areas using ImageJ. * p < 0.01. B. Bar graphs show the percent of the original tumor remaining after either BLS or the combination of BLS + FGS.

Retroperitoneal mouse models of HT-1080-GFP human fibrosarcoma were established in 22 mice (Fig. 2A). One week after retroperitoneal implantation, the mice were randomly divided into the BLS or BLS + FGS groups (11 mice in each group) (Fig. 1). Before surgical resection, the fluorescent-tumor areas of the mice in the BLS and BLS + FGS groups were 41.3 ± 15.4 mm^2^ and 47.9 ± 30.6 mm^2^, respectively (Fig. 2B). There was no significant difference in pre-operative tumor burden between BLS and BLS + FGS mice. A great improvement in visualization of the primary fibrosarcoma during FGS enhanced distinction of tumor from surrounding soft tissues and identified a larger extent of tumor growth due to GFP expression by the tumor (Fig. 3A). FGS following BLS resulted in a more complete resection of the fibrosarcoma, demonstrated by a significant decrease in the fluorescent area of the residual tumor compared to BLS-only. Fluorescent areas of the residual tumors after BLS and the combination of BLS + FGS were 10.5 ± 2.4 mm^2^ and 0.4 ± 0.4 mm^2^, respectively (Fig. 3B; p = 0.00597).

### Time-course imaging of recurrent tumor growth after BLS or the combination of BLS + FGS

**Figure 4:**
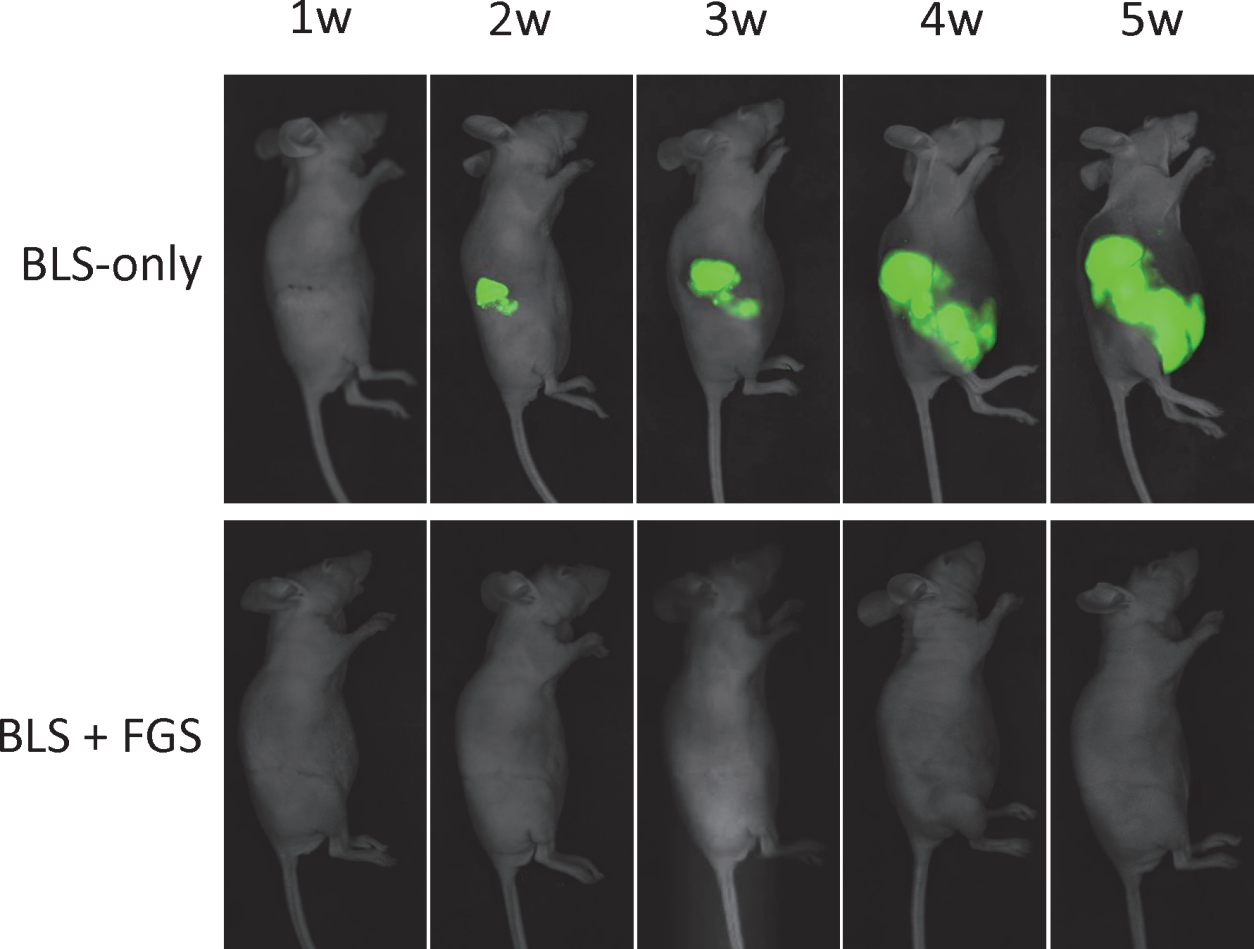
Representative time-course imaging of tumor recurrence after BLS-only and the combination of BLS + FGS. Fluorescence imaging, using the iBOX Scientia Small Animal Imaging System [34, 52, 53], showed BLS-only mice treated had tumor recurrence. In contrast, mice treated with the combination of BLS + FGS showed little recurrent tumor growth.

**Figure 5:**
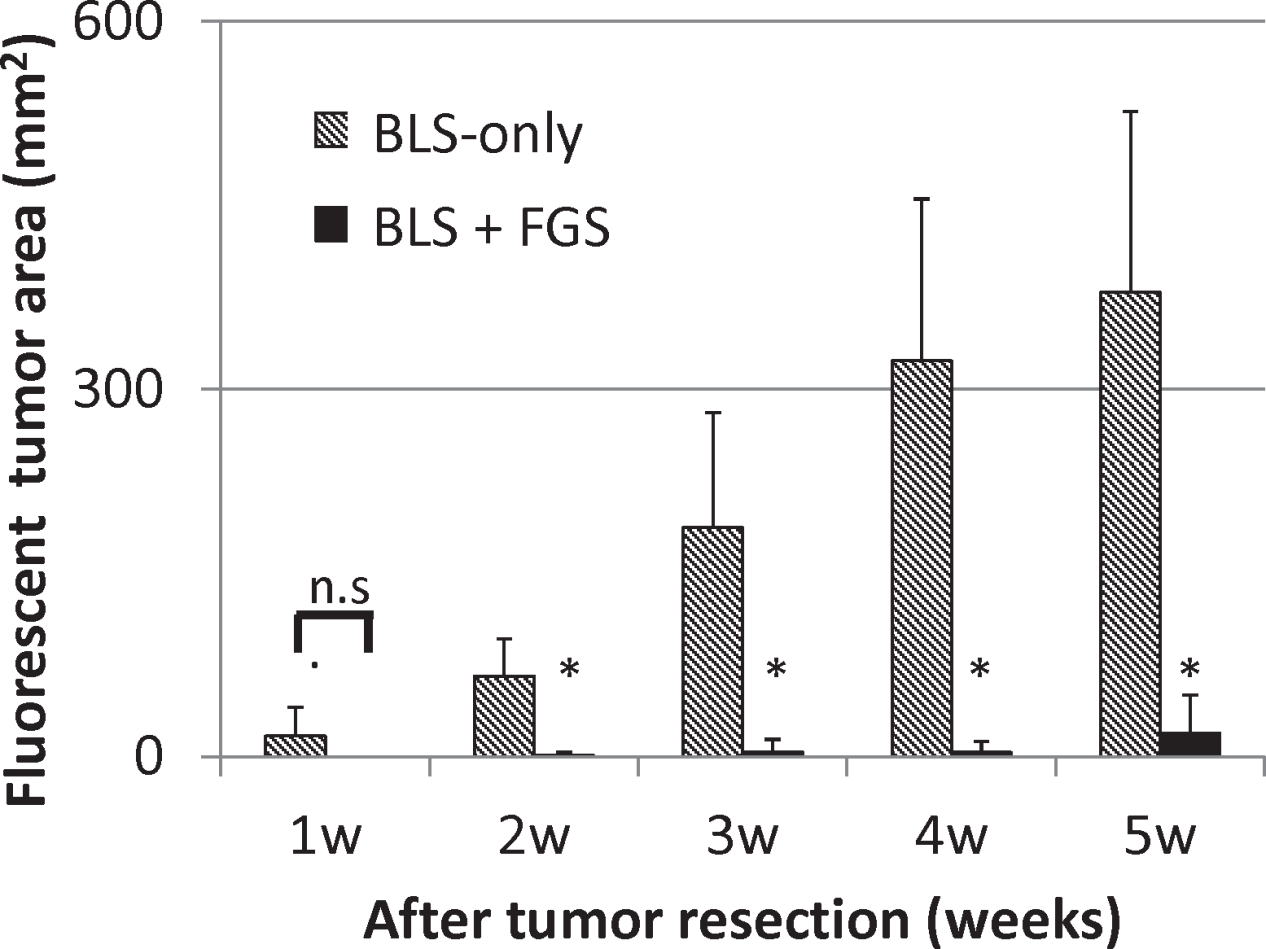
Extent of tumor recurrence after BLS-only or BLS+FGS. BLS-only treated mice had tumor recurrence. BLS + FGS-treated mice had only minimal growth of recurrent tumors. Five weeks after surgery, the fluorescent-tumor areas of BLS-only- and BLS+FGS-treated mice were 379 ± 147 mm^2^ and 11.7 ± 6.9 mm^2^, respectively. The combination of BLS + FGS significantly decreased recurrence compared to BLS-only treated mice. * p < 0.001.

Time-lapse imaging visualized rapid growth of the fluorescent area in the BLS-only-treated mice after surgery (Fig. 4). Fluorescent-tumor areas of the mice treated with BLS-only rapidly increased, whereas mice treated with BLS + FGS had minimal tumor recurrence (Fig. 5). Five weeks after surgery, the fluorescent areas of BLS-only- and BLS + FGS-treated mice were 379 ± 147 mm^2^ and 11.7 ± 6.9 mm^2^, respectively (Fig. 5). The combination of BLS+FGS significantly decreased tumor recurrent more than BLS-only treated mice (p < 0.001).

### Disease-free survival (DFS) after BLS and BLS + FGS

**Figure 6:**
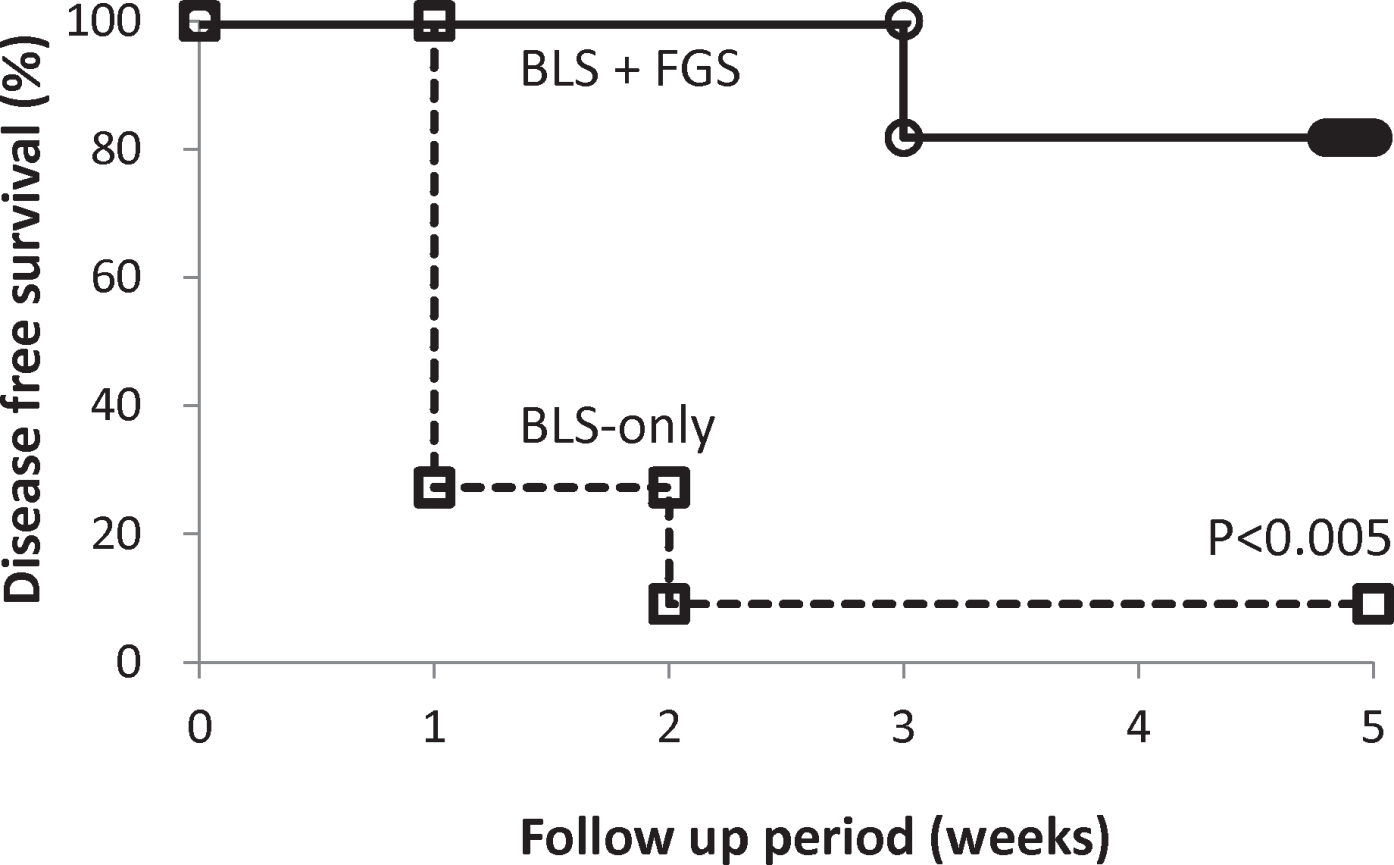
Kaplan-Meier curve for disease-free survival (DFS). Five-week DFS rates of BLS-only and BLS + FGS-treated mice were 9.1% and 81.8%, respectively. Mice treated with BLS + FGS had a significantly higher survival rate than mice treated with BLS-only (p < 0.005).

Five-week DFS rates of BLS-only- and BLS + FGS-treated mice were 9.1% and 81.8%, respectively (Fig. 6). Mice treated with BLS + FGS had a significantly higher survival rate than mice treated with BLS-only (p < 0.005). These results further suggest that FGS significantly reduced the residual tumor volume after BLS and improved DFS.

We have previously demonstrated the enhanced visualization and resection of primary and metastatic cancer labeled with GFP [7–16] or fluorescent antibodies [17–29]. The results of these previous studies show the great potential of FGS. The present study demonstrates that FGS can be beneficial for patients with retroperitoneal sarcoma to prevent recurrence of the tumor, a major current problem in the clinic. A possible translatable method to label the fibrosarcoma in the clinic could be with a GFP-containing, telomerase-dependence adenovirus which effectively and selectively labels tumors in mouse models, enabling FGS [7–9]. Another possibility could be the use of RNA-guided nano-sensors to specifically label cancer cells in vivo for FGS [42].

## Supporting Information

S1 ARRIVE Checklist. (PDF)
